# Music and Language in the Human Brain: Mismatch Negativity Evidence for Their Neuroplasticity and Interplay

**DOI:** 10.1111/ejn.70399

**Published:** 2026-01-21

**Authors:** Mari Tervaniemi

**Affiliations:** ^1^ Centre of Excellence in Music, Mind, Body, and Brain, Department of Education, Faculty of Educational Sciences University of Helsinki Helsinki Finland; ^2^ Cognitive Brain Research Unit, Department of Psychology, Faculty of Medicine University of Helsinki Helsinki Finland

**Keywords:** auditory learning, MMN generators, preattentive processing, predictive coding

## Abstract

We live in an auditory world; we perceive and memorize sounds even before birth. Here, I have reviewed findings on the effects of implicit and explicit learning on auditory neurocognition. These findings, investigated in the mismatch negativity (MMN) paradigm, indicate that both implicit and explicit auditory expertise can modulate the neural encoding of sounds at the preattentive level of human cognition. This modulation is reflected in the MMN parameters latency and amplitude and its generators. Thus, studies on MMN can illustrate various forms of human auditory learning during development and learning across the entire lifespan.

## Graphical abstract

While several empirical findings and influential theories promote the importance of the left auditory cortex in language encoding and the corresponding areas in the right hemisphere in music encoding, there are also some intriguing findings opposing these views. Some of those originate from the literature reviewed and discussed in this paper.

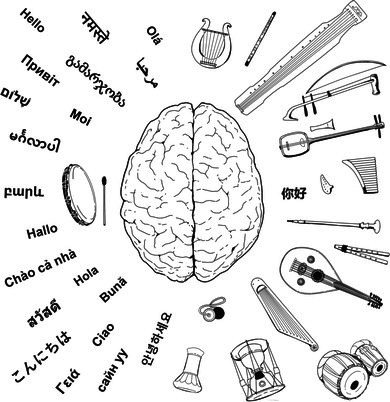

**Abbreviations**
EEG: electroencephalogram
fMRI: functional magnetic resonance imaging
MEG: magnetoencephalographic
MMN: mismatch negativity
PET: positron emission tomography
TMS: transcranial magnetic stimulation

## 1 Introduction

Speech and music are among the key forms of human cognition and interaction and are generally used frequently. Speech is used for communication, and music is used for listening. Music, such as singing or playing a musical instrument, can also be a hobby or professional activity, thus providing an additional means to convey emotions. Acoustically and cognitively, speech and music share various principles, such as a hierarchy from single items (i.e., a chord or phoneme) to complex entities (i.e., melodies or sentences). Neurally, both speech and music are processed along the auditory pathways from the cochlea via the midbrain up to the auditory cortices and association areas in the left and right hemispheres.

In this review, I will introduce findings from various eras of research within auditory cognitive neuroscience in which speech and music functions and learning were the study focus. My focus will be on the findings based on studies using mismatch negativity (MMN) as a probe for human auditory neurocognition, learning and neuroplasticity. Originally, MMN was interpreted as a neural index of short‐term memory of acoustical sound features (Näätänen and Alho 1995). Later, however, higher order abstract sound features were also considered as part of preattentive cognition (Näätänen et al. 2001; Paavilainen 2013). Finally, the commonalities and iterative processes between short‐ and long‐term memory were identified, and the interpretations regarding MMN elicitation were expanded to also cover the effect of long‐term memory in MMN elicitation (Schröger et al. 2004; Näätänen et al. 2010). In this framework, the pioneering findings on the effects of implicit and explicit learning on MMN were and are of great importance. Most recently, MMN interpretation has been considered within the predictive coding framework (for an overview, see Friston 2005; for MMN interpretation Winkler 2007; Schröger et al. 2014; Fitzgerald and Todd 2020). The predictive coding framework is of great importance for the speech and music literature. Indeed, their content must be predicted with ease and accuracy; otherwise, fluent communication and interaction via these two domains would fail.

## 2 MMN Amplitude as an Index of Neuroplasticity in Music and Language

The earliest evidence for learning speech and music in the developing brain can be observed already immediately after birth. This was shown as an enhanced mismatch response in newborn babies whose mothers played speech and music CDs to their foetuses while pregnant (Partanen et al. 2013). One speech sequence on the CD was a pseudoword ‘tatata’; this was presented as ‘taTAta’ during the recordings, with the middle syllable having a higher pitch and evoking a larger mismatch response in the newborn babies of the experimental than control group (mothers who did not play these CDs while pregnant). Along these same lines, and by using the MMN paradigm, Kostilainen et al. (2020) showed that even babies born prematurely (mean 30.5 gestational weeks at birth) can neurally discriminate between acoustically and emotionally different speech sounds; the recordings were conducted when the babies reached 40 gestational weeks and their mismatch responses were highly similar to those of full‐term babies. Furthermore, this discrimination was enhanced by parental singing and humming directed toward the newborns during their care (Kostilainen et al. 2021). Virtala et al. (2023) showed that in infants with an inherited dyslexia risk, an intervention with music listening at 0–6 months resulted in enlarged MMN responses as determined at the age of 6 and 28 months. This comparison was made between infants with an intervention with vocal music, instrumental music and without any intervention; MMN enhancement was selectively found in the babies who were in the vocal music group. Finally, François et al. (2017) found that mismatch responses obtained from newborns showed successful discrimination of deviants in the musically enriched but not in the ‘flat’ sound condition. Moreover, a year and a half later, an association was found between the mismatch responses (as obtained immediately after birth) and the child's expressive vocabulary. Thus, auditory learning occurs very early in human development, is affected by music enrichment, and can be investigated by MMN (and other ERP) recordings before any overt behavioural methods can be used.

Later in development, musical training and exposure can improve various speech and language functions, such as pronunciation, reading skills, phonemic encoding and memory (for reviews, see Milovanov and Tervaniemi 2011; Gordon et al. 2015; Linnavalli et al. 2021). These findings indicate that musical expertise and exposure can also be associated with skills and behaviour not directly related to music functions. There is also MMN evidence available for some of these behavioural findings. For instance, participants with more advanced skills in foreign‐language pronunciation (who also have higher scores in a musicality test) have enhanced MMN to musical and speech sounds (Milovanov et al. 2008, 2009). MMN is enhanced in musicians (Koelsch et al. 1999) and can reflect the specific aspects of their musical training in terms of the most common genre in their repertoire (Vuust et al. 2012; Tervaniemi et al. 2016). For reviews on the effects of music training in audition, see Herholz and Zatorre 2012; Putkinen and Tervaniemi 2018; and Putkinen et al. (Forthcoming).

In a similar vein, language background can modulate preattentive auditory processes as indexed by MMN. The pioneering study by Näätänen et al. (1997) showed how phonemes of one's native language are predominantly discriminated in the brain, and this processing even overrules the acoustical dissimilarity between the standard and deviant phonemes. In addition to such modulatory effects of native language exposure to phoneme processing, MMN to nonspeech sound duration changes was enhanced in Finnish quantity‐language speakers when compared with speakers of other languages (Ylinen et al. 2006; Tervaniemi, Jacobsen, et al. 2006; Marie et al. 2012). In quantity languages, the phoneme duration denotes semantic meaning; for instance, in Finnish, tuuli (wind) and tuli (fire) or pako (escape) and pakko (forced) differ from each other only in the length of /u/ or /k/. In all these studies, conducted in German, Russian, and French speakers in addition to Finnish speakers, MMN to other deviances such as pitch did not differentiate the speakers based on their different native languages. This implies that the sensitization to discriminate duration cues was specific to that semantically important sound feature without generalized facilitation related to Finnish language as such.

The most recent investigations along this line of research were performed with speakers of the Estonian language (Lyu et al. 2024a). Estonian speakers denote semantic meaning via duration cues that are more advanced than Finnish, with three phonetic durations (short, long and over‐long). They are further supported by pitch cue, particularly to help differentiation between long and over‐long phonemes. In a MMN paradigm with speech and nonspeech stimulation, Estonian and Chinese speakers were compared in their ability to differentiate between original sounds and their pitch, duration or pitch‐ and duration‐modulated variants. The main results indicated that Chinese speakers had larger MMN responses than Estonian speakers to pitch modulations in the Chinese‐based stimuli. In addition, Estonian speakers had larger MMN responses than Chinese speakers to the nonspeech pure tones of the Estonian stimuli that contained both duration and pitch changes. In a parallel study, Lyu et al. (2024b) also found evidence for the effect of musical expertise on speech sound processing using behavioural and MMN paradigms. A larger MMN among Chinese and Estonian musicians than among nonmusicians was found for the simultaneous duration and pitch change when it was presented in their native languages. When sound changes were presented in their non‐native languages, both Chinese and Estonian musicians were more accurate in attentional discrimination than nonmusicians; however, no MMN evidence for the musicians' superiority was found. Thus, as reflected by the MMN, musicianship seems to be in a closer relationship with native language processing than with non‐native language processing.

Regarding the possible drivers behind MMN enhancement in the studies described above, it is worth noting that this could reflect learning‐related mechanisms at both sensory and predictive levels that operate in complementary ways. At the sensory level, experience may sharpen auditory representations, thereby increasing discrimination accuracy. At the predictive level, learning could increase the precision of internal models, leading to stronger prediction–error responses.

## 3 Longitudinal Evidence on MMN as an Index of Interaction Between Speech and Music Processing in the Brain

It is worth gaining familiarity with the cross‐sectional studies in adults, such as those introduced above. However, to find causal relationships in human learning, longitudinal follow‐up studies are of special interest and value. Previously, such studies have been conducted, e.g., by Moreno et al. (2009) and Chobert et al. (2014), who showed that music training can enhance neural pitch discrimination. Furthermore, Frey et al. (2019) showed that music training can also enhance the MMN to voice‐onset changes among participants with dyslexia (for a review, see Besson et al. 2007).

Recently, we showed in a comparable study design that foreign‐language training modulated the participants' auditory brain responses, particularly the pitch MMN of a melodic multifeatured MMN paradigm (Tervaniemi et al. 2022). In that study, over 100 Chinese school children aged 8–11 years were recruited into a longitudinal intervention study of one academic year. Some children were given music lessons, and some children were given lessons in English. These lessons took place after the regular school day in a group setting. As the passive control group, we had children who participated in a regular school programme without any additional activities. Our hypothesis about music lessons enhancing music‐specific MMN was not confirmed. Instead, we observed that English lessons enhanced the pitch MMN in a musical (melodic) paradigm more than music lessons. This puzzling but promising finding may reflect the primary sensitivity present in tonal‐language speakers to encode new auditory (even speech) information as musical information. Accordingly, this finding might be restricted to preattentive auditory functions of tonal‐language speakers only and should not yet be generalized to other language speakers or to behavioural listening and pronunciation skills.

## 4 MMN as an Index of Hemispheric Asymmetry in the Brain

I will now move from electroencephalogram (EEG)‐based MMN studies to studies in which speech and music functions were investigated by using brain‐mapping methods with more advanced means of localizing the source of the MMN generator(s). When using EEG, the MMN is classically predominant at the right‐hemispheric frontocentral electrodes. By dipole modelling, the generators of these MMN responses have also been localized into the auditory cortices and, in some studies, into the right frontal cortex (Rinne et al. 2000; Opitz et al. 2002; for a pioneering review, see Alho 1995). Recently, it was shown that the MMN sources for acoustic deviances (pitch, timbre and timing) can be differentiated from those for musical (more cognitive) deviances (transposition, melodic contour and rhythmic patterns) when all these six deviances were introduced in the same recurrent 2‐s melody (Bonetti et al. 2022). Based on magnetoencephalographic (MEG) recordings and inverse modelling of the data obtained, the acoustic deviances were processed at the primary and secondary auditory cortices, while cingulate and orbitofrontal cortices were active for discriminating the cognitive deviants. For other studies that used the melodic multifeature paradigm, see Tervaniemi et al. (2016) and Vaquero et al. (2021).

As mentioned above, brain‐mapping methods that allow more fine‐tuned localization include magnetoencephalogram (MEG, with excellent temporal resolution), functional magnetic resonance imaging (fMRI, with excellent spatial resolution but high‐level acoustic scanner noise) and positron emission tomography (PET, with good spatial resolution without background noise as in fMRI). The aim of these early empirical efforts was to indicate whether the hemispheric (right‐predominant) asymmetry in the MMN generation is preserved in speech and music sound encoding or whether the MMN generator could reflect the speech versus music asymmetry also in the healthy brain (as suggested by lesion studies; see below).

In general, the brain hemispheres are not identical regarding music and speech processing. Based on early observations on patients with brain lesions, the basic principles of brain asymmetry have been known since the early 1900s. Lesions of the left temporal lobe disturb speech functions, while lesions of the right temporal lobe disturb music functions. However, only in recent decades has research been able to investigate the brain basis of this asymmetry in a healthy brain (see Hickok and Poeppel 2007; Tervaniemi and Hugdahl 2003; Zatorre et al. 2002).

**FIGURE 1:**
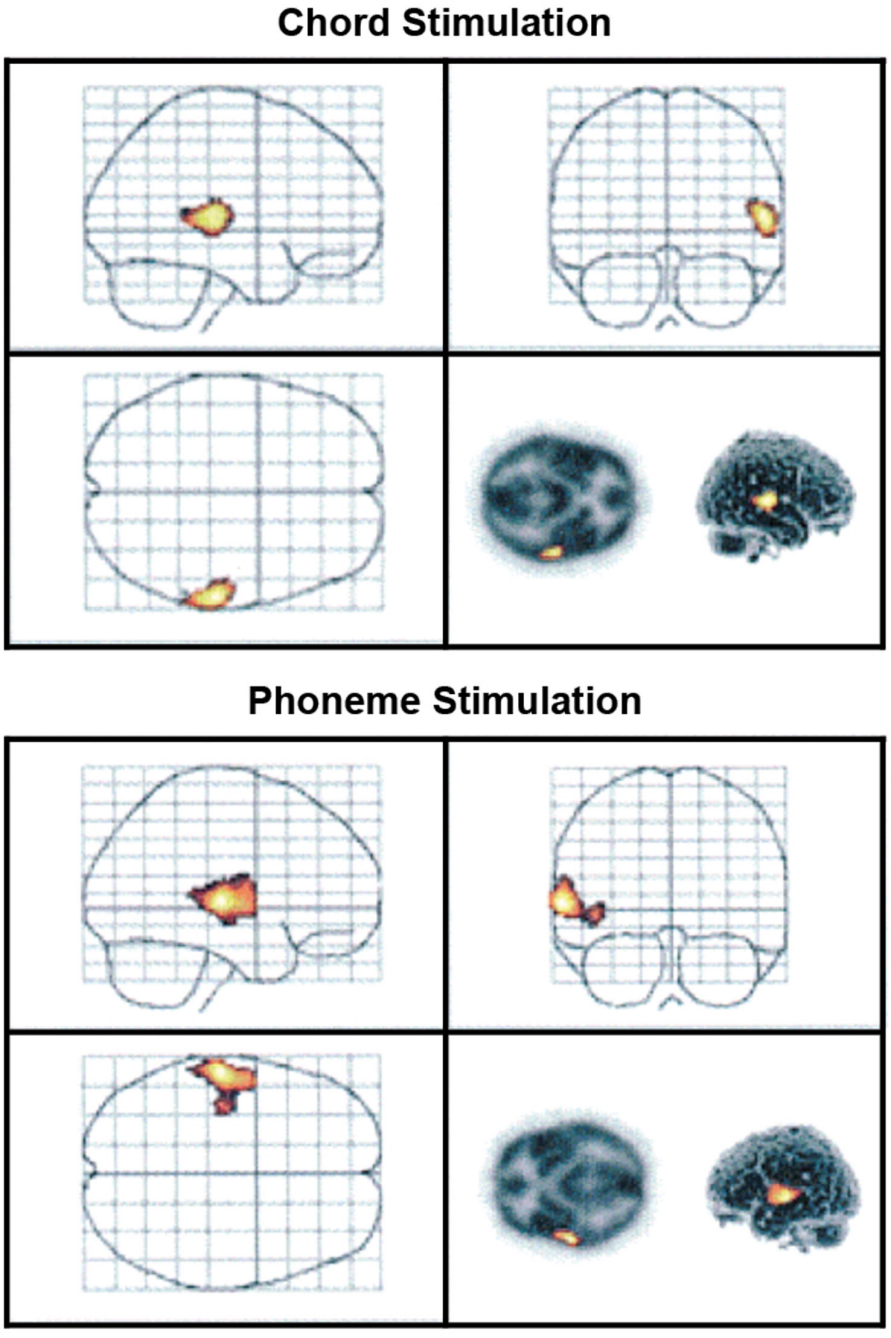
The activated areas in deviant and standard minus standard comparison. Upper panel: Activated area in the right superior temporal gyrus during chord stimulation. Lower panel: Activated area in the left superior and middle temporal gyri during phoneme stimulation. Figure originally published in Human Brain Mapping, 2000 by Tervaniemi et al. Reproduced with permission obtained from the publisher.

First, to investigate the brain basis of discrimination of slight changes in music and speech sounds, we showed that, as reflected by the MMN, the earliest stage of this discrimination is asymmetric according to traditional views. Chord processing occurs predominantly on the right and phoneme processing predominantly on the left hemisphere. This conclusion was reached by pioneering MMN studies using MEG recordings and PET scanning (Tervaniemi et al. 1999; Tervaniemi, Medvedev, et al. 2000). In both studies, sequences of phonemes and chords were presented to participants, while they were instructed to focus on a task unrelated to the sound stimulation. In Tervaniemi, Medvedev, et al. (2000) (data illustrated in Figure 1), the task was to indicate via a button press the grammatical gender of nouns presented on a visual display (due to technical reasons, it was not possible to use muted video as a primary task). The sequences consisted of either standard phonemes or chords (100% probability each) or included both standard (85%) and deviant (15%) phonemes or chords. Even if several acoustic features differed between phonemes and chords, in our stimulation we balanced the acoustic difference between standard and deviant in both stimulus types. It was possible by matching the acoustical difference between them to be of one octave in magnitude using phonemes/e/versus /o/ and chords A major versus A minor (other acoustical features, such as stimulus duration, presentation rate and intensity were also matched between the phonemes and chords). This implies that the hemispheric asymmetry in processing musical and phonetic stimulation shown in several attentive paradigms is caused by automatically activated lateralized neural mechanisms that can be determined in the MMN measurements.

Second, it is worth noting that in addition to phonemes and major or minor modes, language and music also contain finely grained acoustic cues that denote, for instance, prosody (emotional or linguistic prosody in speech), changes in tempo (shortened or prolonged tone durations) or changes in intensity (implying changes in emotional connotations of music). In the second wave of research to compare language and music in the brain, we took this level of sound information into our focus (Tervaniemi, Szameitat, et al. 2006). To go one step further from isolated phonemes and chords, we aimed to use pseudowords pronounced by professional actors and the musical counterparts of these pseudowords. As it was observed that saxophone sounds are very similar to the human voice in their spectra, the study was implemented using pseudowords (baba) and a corresponding digital counterpart in pitch and duration based on a sample of a saxophone sound. To go one step further also in brain scanning technology, the study was implemented using 3‐T fMRI in a sparse‐sampling paradigm. Similar to the MEG and PET studies described above, there were sequences with standards only and with standard and deviant sounds intermixed. The exact sound stimulation parameters were chosen after conducting an extensive MMN study using several EEG recording sessions in which musicians and nonmusicians in ignore and attend conditions were compared (Tervaniemi et al. 2009). As the result of this paradigm optimization, the fMRI study was launched with nonmusicians as participants in a semi‐ignore condition; the participants were instructed to indicate by a button press whether they heard speech or music sounds. They were not informed about the existence of slight duration and pitch deviants that were of identical magnitude in speech (pseudowords) and music (saxophone sound) sequences.

**FIGURE 2:**
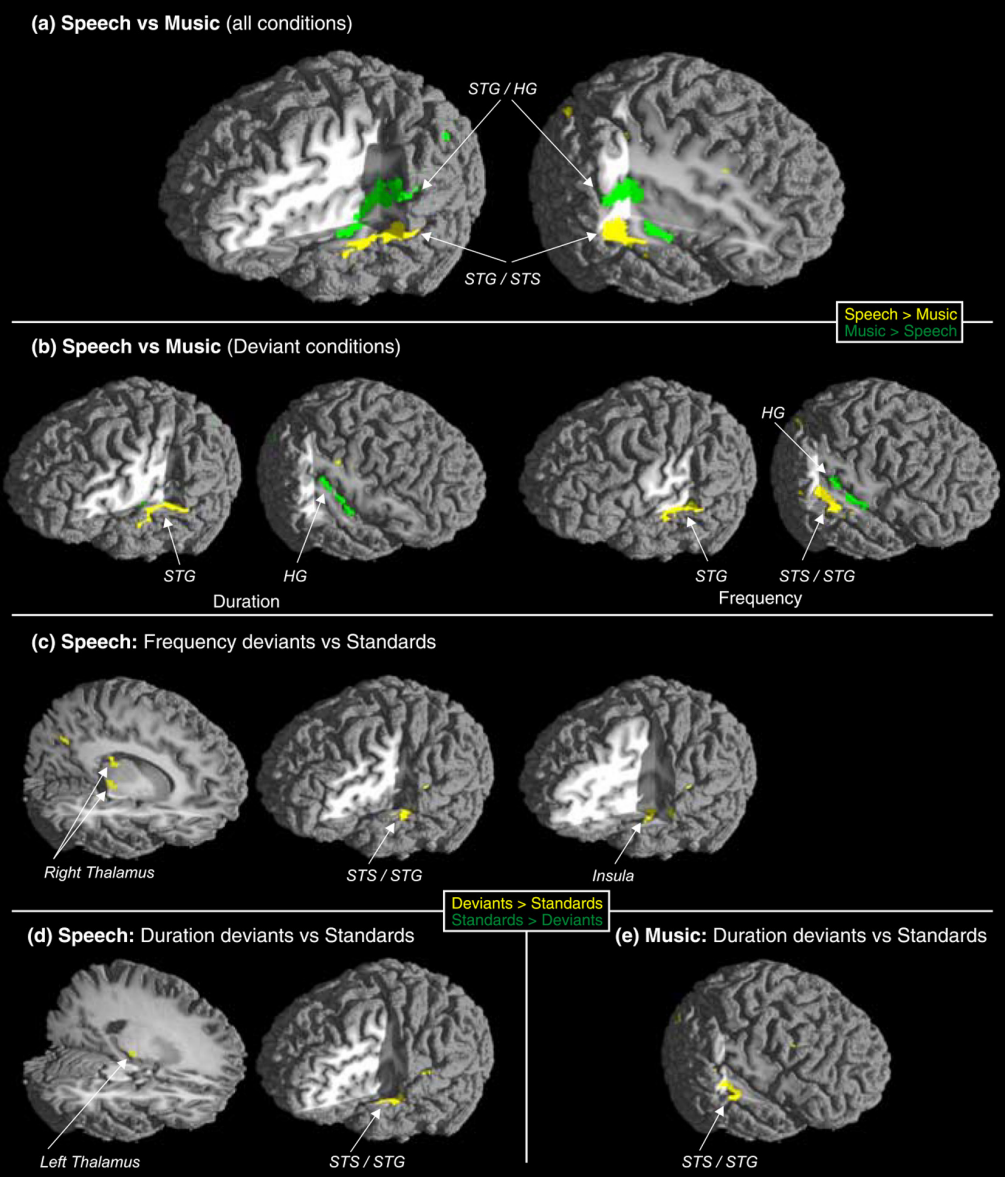
Significant foci of activity in speech versus music sound contrast (all conditions included) (a), speech versus music deviant contrasts (b), deviant versus standard contrast for speech (c,d) and deviant versus standard for music (e). (a,b) Yellow colour illustrates stronger activation for speech than music sounds, and green colour illustrates stronger activity for music than speech sounds. (c–e) Yellow colour illustrates stronger activation for deviant than standard sounds, and green colour illustrates stronger activity for standard than deviant sounds. Figure originally published in Journal of Neuroscience, 2006, by Tervaniemi et al. Reproduced with permission obtained from the publisher.

The results are shown in Figure 2 and can be summarized as follows: The areas encoding music and speech sound changes differed in the cortical and subcortical areas. In the temporal lobe, music sounds were processed in sources more medial than sources for speech sounds. Further, asymmetric thalamic activation caused by pitch and duration deviants was observed with speech sounds but only cortical activation with music sound deviants. It thus seems that even the most elementary stages of sound encoding and discrimination can differ between sound categories (speech/music) and also between sound parameters (pitch/duration). As the participants were nonmusicians, with substantial experience in hearing and producing speech daily but less so with music, we might speculate that the thalamic asymmetry reflects the participants' familiarity with speech encoding. Note that the data were collected in early 2000 when constant music listening was uncommon due to lack of mobile technology and streaming services, implying that the difference in speech versus music exposure in these musically nontrained participants was more evident than it would be in current circumstances when implicit music learning is very common.

## 5 Interlude to Literature Outside MMN

Based on the MMN evidence above, it was an open question whether speech and music are encoded in the human cortical (and subcortical) areas in an asymmetric manner due to the informational content of the sound material or due to the acoustic parameters that are most dominant in speech and music. As suggested by early reviews (see Hickok and Poeppel 2007; Tervaniemi and Hugdahl 2003; Zatorre et al. 2002), the asymmetry might reflect different roles and specializations of the brain hemispheres in processing sound features that are dominant in speech and music, namely, fast temporal information intrinsic to speech and fine‐tuned pitch information intrinsic to music. Using fMRI and parametric manipulation of noise‐like sound sequences (Schönwiesner et al. 2005), time‐ and frequency‐modulated sinusoidal ripples (Schönwiesner and Zatorre 2009), or sentences and songs (Albouy et al. 2020), it was indeed shown that left‐hemispheric activity is predominant when modulating temporal (time) information and right‐hemispheric activity when modulating spectral (pitch) information. These findings provide strong support for the views according to which the key sound features within each domain (temporal in speech and spectral in music) underlie the observed brain asymmetry rather than the domain (speech or music) itself.

## 6 Can Brain Asymmetry in Audition Be Modulated by Long‐Term Expertise?

As discussed above, the brain hemispheres seem to be adjusted to feature‐specific processing of auditory information and not speech and music as such. Notably, however, this feature‐specific asymmetry is not necessarily fixed but, in fact, might be subject to neuroplasticity and long‐term auditory learning. Although tentative evidence on this comes from cross‐sectional studies on adult participants and should be interpreted with caution, it offers a very promising starting point for further longitudinal and intervention studies.

Regarding chord discrimination in a paradigm adopted from Tervaniemi et al. (1999) and Tervaniemi, Medvedev, et al. (2000), we observed predominantly right‐lateralized MMN responses on musically naïve participants (MEG evidence in Tervaniemi et al. 2011). However, we observed bilateral MMN responses in musically trained participants and in nonmusicians scoring well in a musicality test (Tervaniemi et al. 2011). In a similar vein, rhythm discrimination evoked right‐dominant MMN in nonmusicians but left‐lateralized MMN in jazz musicians (Vuust et al. 2005).

Correspondingly, in language studies, Gu et al. (2013) observed left‐dominant MMN for semantic language‐specific discrimination and more bilateral MMN for acoustic discrimination. All participants in this study were Chinese speakers, meaning that in their language, pitch‐variant contour of the words carries semantic meaning (i.e., they are tonal‐language speakers). Further, by using magnetic pulses (transcranial magnetic stimulation, or TMS) to disrupt the part of the motor cortex serving speech functions, Tang et al. (2021) showed that tonal‐ and nontonal‐language speakers differ in MMN if their speech‐related motor functions are disturbed; the disturbance of the right speech motor cortex suppressed responses of the auditory cortex to tone changes in nontonal‐language speakers. In contrast, the disturbance of the left speech motor cortex suppressed responses to tone changes in tonal‐language speakers. For phoneme changes, disruption of the left but not right speech motor cortex suppressed responses in both language groups.

Taken together, the evidence from music and language studies indicates that the hemispheric asymmetry in audition is not caused only by the sound material being either music or speech but that it rather reflects the sound features that dominate in each of these modes of sound. Further, the evidence also suggests that the hemispheric asymmetry in audition is subject to neuroplasticity and learning.

## 7 Methodological Considerations

In the earliest MMN studies, experimental sound sequences consisted of two different sounds both consisting only of sinusoidal (pure) tones, namely, frequently presented standards and rarely presented deviants (e.g., probabilities 0.9 and 0.1, respectively; e.g., see Sams et al. 1985).

However, thanks to theoretical and methodological development, more complex (and ecologically valid) paradigms were developed, such as the so‐called multifeature paradigm used by Kostilainen et al. (2021) in their study on newborn infants. The paradigm had one standard (with a probability of 0.46) and a total of nine different deviants. The pseudoword/ta‐ta/served as a standard sound being interspersed with the following deviants: vowel duration/ta‐ta:/, vowel change/ta‐to/, intensity change ±6 dB and frequency changes ±25.5 Hz; emotional deviant stimuli were/ta‐ta/, which expressed happy, sad and angry emotions. This paradigm, originally developed by Pakarinen et al. (2007) on the initiative by Professor Näätänen, is highly cost‐effective regarding time needed for data collection without compromising data quality.

Correspondingly, as cited above, musically relevant paradigms have also been developed based on chord arpeggio (Vuust et al. 2012) and melody repetition (Tervaniemi et al. 2016). In parallel, the sound structures in MMN studies often resemble real‐life sounds, with several harmonic partials (overtones) in otherwise simple (Tervaniemi, Schröger, et al. 2000) or more advanced (Tervaniemi, Jacobsen, et al. 2006) paradigms. By these means, the ecological validity of the paradigms has been considerably improved.

Mobile EEG technology may better mimic real‐life contexts for MMN studies. Current technology in electrodes and amplifiers enables recordings without an electrically shielded cabin (Faraday cage). For participant comfort and the possibility of participating without travelling and adjusting to new surroundings, this is an important improvement (Tervaniemi 2025). Previously, MMN and P3a recordings using oddball‐ and multifeature paradigms were successfully conducted in day care centres (Linnavalli et al. 2017, 2018; Byman et al. 2025), schools (Moreno et al. 2009; Widmann et al. 2012; Linnavalli et al. 2022), and in an office space (Slater et al. 2014; see also a review by Bradley et al. 2025 for use of mobile EEG technology in music and speech studies in infants).

## 8 Discussion and Conclusions

Speech and music, which are the most essential forms of daily auditory cognition and interaction, rely on sequential processing in the brain from the cochlea until the auditory cortices and beyond. In this review, I have highlighted the key functions of this processing in the MMN framework, by first introducing MMN as a functional index of processing efficacy and then by describing MMN asymmetry as an index of the processing within each auditory cortex.

In both parts of this review, the main emphasis has been on the neuroplasticity of the processes behind MMN elicitation. Learning, whether implicit or explicit, has its imprints on the brain that can be observed via MMN recordings. It is worth noting that in speech and music domains, both implicit and explicit forms of learning take place; native language can be learnt up to a great extent implicitly, while foreign languages, in most cases, need explicit learning to be mastered. Learning to play a musical instrument requires explicit learning (with or without a teacher), while certain aspects of one's own musical culture are implicitly learnt. Interestingly, singing, which combines both speech and music as one entity, can be learnt both implicitly and explicitly.

Furthermore, as tentatively shown by now, it is likely that the domains of expertise in audition (namely, in language and music) also interact with each other. This is a subject for future studies. If these studies seek to investigate the functions of the MMN without emphasis on the neuroanatomical sources of it, then one might consider the use of mobile EEG technology. While reliable source modelling of MMN activity is then less prominent, on the other hand, participant recruitment is more likely to succeed in reaching participants with expertise in music or in the languages the study is focused on (Tervaniemi 2025).

In summary, the MMN literature has developed remarkably in recent decades. Originally, in the 1980s, MMN was considered to reflect short‐term memory traces. However, as the current review illustrates, MMN can also reflect highly abstract auditory entities and long‐term memory representations. These representations provide the basis of auditory predictions that are of high relevance to music and speech encoding and thus to auditory communication. Since the MMN can be recorded in the ignore condition, this allows monitoring of various forms of learning without concomitants and interference caused by differences in motivation or anxiety that may occur during overt task performance in many participants. Furthermore, as also discussed above, the stimulation paradigms are approaching ecological validity (Tervaniemi 2022; Tervaniemi 2023), and the recording setups include, in addition to lab‐based high‐density EEG, MEG and brain‐mapping techniques, mobile EEG for recordings in day cares, schools and workplaces. Consequently, MMN, with a long history at the crossroads of human perception and action in audition, will continue to provide a convenient means of investigating human auditory neurocognition and its plasticity across the entire lifespan.

## Author Contributions

**Mari Tervaniemi:** conceptualization, funding acquisition, methodology, writing – original draft, writing – review and editing.

## Funding

The study was funded by the Research Council of Finland.

## Conflicts of Interest

The author declares no conflicts of interest.

## Data Availability

This review is based on empirical studies already published and cited in this paper.
